# Supplementary material for: Conditional chemoconnectomics (cCCTomics) as a strategy for efficient and conditional targeting of chemical transmission
Source: eLife. 2024 Apr 30;12:RP91927. doi: 10.7554/eLife.91927 (PMC11060718; doi:10.7554/eLife.91927)
Supplement: Figure 3—source data 3. [file elife-91927-fig3-data3.zip › Figure 3 source data3 part 1.zip!/Figure 3-Source data 3-part1/1071-1-CRISPResso_Report_osfbjvga.zip!/CRISPResso_on_1071_2/1c.Alignment_barplot.pdf]

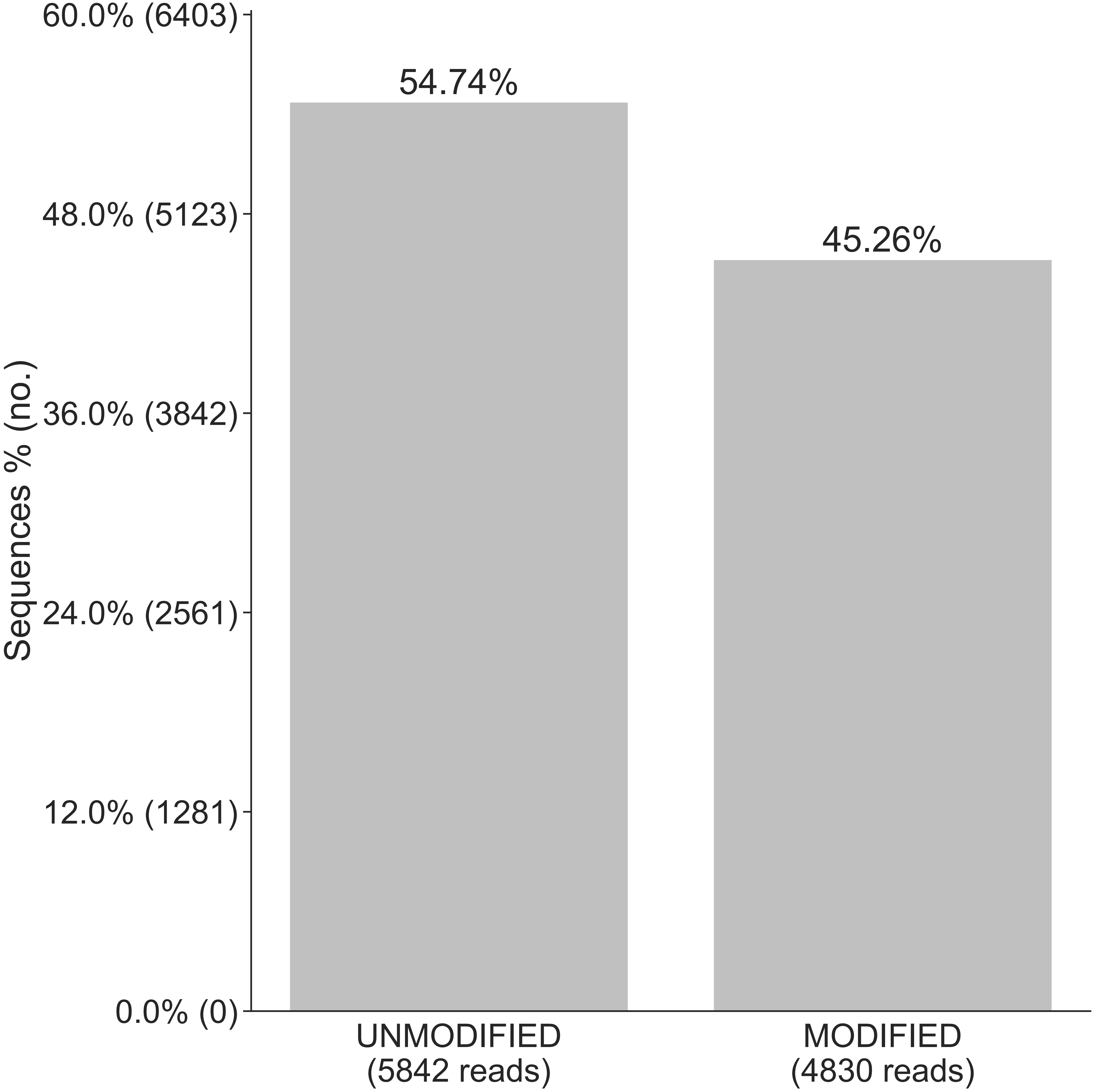

Sequences % (no.)
60.0% (6403)
48.0% (5123)
36.0% (3842)
24.0% (2561)
12.0% (1281)
0.0% (0)
54.74%
45.26%
UNMODIFIED
(5842 reads)
MODIFIED
(4830 reads)
